# PATO: genome-wide prediction of lncRNA–DNA triple helices

**DOI:** 10.1093/bioinformatics/btad134

**Published:** 2023-03-16

**Authors:** Iñaki Amatria-Barral, Jorge González-Domínguez, Juan Touriño

**Affiliations:** Computer Architecture Group, Department of Computer Engineering, CITIC, Universidade da Coruña, Campus de Elviña, A Coruña 15071, Spain; Computer Architecture Group, Department of Computer Engineering, CITIC, Universidade da Coruña, Campus de Elviña, A Coruña 15071, Spain; Computer Architecture Group, Department of Computer Engineering, CITIC, Universidade da Coruña, Campus de Elviña, A Coruña 15071, Spain

## Abstract

**Motivation:**

Long non-coding RNA (lncRNA) plays a key role in many biological processes. For instance, lncRNA regulates chromatin using different molecular mechanisms, including direct RNA–DNA hybridization via triplexes, cotranscriptional RNA–RNA interactions, and RNA–DNA binding mediated by protein complexes. While the functional annotation of lncRNA transcripts has been widely studied over the last 20 years, barely a handful of tools have been developed with the specific purpose of detecting and evaluating lncRNA–DNA triple helices. What is worse, some of these tools have nearly grown a decade old, making new triplex-centric pipelines depend on legacy software that cannot thoroughly process all the data made available by next-generation sequencing (NGS) technologies.

**Results:**

We present PATO, a modern, fast, and efficient tool for the detection of lncRNA–DNA triplexes that matches NGS processing capabilities. PATO enables the prediction of triple helices at the genome scale and can process in as little as 1 h more than 60 GB of sequence data using a two-socket server. Moreover, PATO’s efficiency allows a more exhaustive search of the triplex-forming solution space, and so PATO achieves higher levels of prediction accuracy in far less time than other tools in the state of the art.

**Availability and implementation:**

Source code, user manual, and tests are freely available to download under the MIT License at https://github.com/UDC-GAC/pato.

## 1 Introduction

Long non-coding RNAs (lncRNAs) are non-protein coding transcripts with more than 200nt in length. Even though lncRNAs were first regarded as transcriptional noise, studies have proved that they play crucial roles in important biological processes. Indeed, lncRNAs regulate gene expression by interacting with chromatin and recruiting chromatin modifiers (Fatica and Bozzoni 2014). lncRNAs chromatin interaction mechanisms include: RNA–DNA hybridization via triplexes, cotranscriptional RNA–RNA interactions, and RNA–DNA binding mediated by protein complexes.

Among these binding mechanisms, the characterization of lncRNA–DNA triple helices has so far been studied in less detail. As a consequence, not many tools have been developed for the *in silico* detection of triplex-forming lncRNA–DNA pairs. Some of these tools, despite providing good levels of prediction accuracy and flexible execution options that allow them to better fit a particular study (Antonov et al. 2019), have nearly grown a decade old and depend on legacy software to run. What is worse, now that RNA–DNA triple helices have been spotted as underexplored avenues for biotechnological and gene therapeutic applications (Hu et al. 2017), new triplex-centric pipelines have started to emerge based on this set of tools that can hardly keep up with the raw processing power of next-generation sequencing (NGS) technologies.

In this article, we present PATO, a modern application that enables the efficient and comprehensive prediction of lncRNA–DNA triple helices at the genome scale. Besides providing genome-wide and NGS-matching processing capabilities, PATO’s efficiency allows a more exhaustive search of the triplex-forming solution space, and so it can predict more accurate triplexes in far less time than other tools in the state of the art.

## 2 Implementation

PATO is a C++17 application that follows the same triplex prediction algorithm as Triplexator (Buske et al. 2012). However, PATO implements a faster and more efficient search of all maximal triplex-forming oligonucleotides (TFOs), triplex target sites (TTSs), and triplexes. We chose Triplexator as the starting point for our work as it showed to be more accurate than other counterparts in a recent study (Antonov et al. 2019). To identify all maximal TFOs and TTSs, PATO uses a finite state automata to list initial candidate regions containing *C* consecutive errors at most. Afterwards, the set of candidate regions is investigated to identify all maximal features using a bounded search that considers both guanine and error rates. To identify all maximal triplexes, PATO exhaustively tests all pairwise combinations of TFOs and TTSs. For each candidate pair $P_{ij}$, PATO extends the TFO–TTS interacting region of $P_{ij}$ using the X-Drop algorithm and subsequently verifies it using a bounded search in order to obtain the set of maximal triplexes derived from $P_{ij}$. We refer to Buske et al. (2012) for a more detailed explanation of the algorithm.

PATO features systematic OpenMP parallel processing capabilities and fine-grained control over the memory usage of its search algorithms, and so it can scale genome-wide analyses from the standard desktop computer to a high-end, multiple-socket server. We consider PATO parallel processing capabilities to be systematic as they successfully accelerate all the steps involved in the identification of triple helices, whereas tools like Triplexator only allow the user to choose from a limited set of parallel run modes that do not accelerate the entire triplex search procedure. PATO allows fine-grained control over its memory usage by providing the user with a command line option to set the maximum number of sequences that may be processed simultaneously (i.e. less simultaneous sequences imply less memory usage). Rather than loading all the sequences in the input files and searching for triplex features over all of them at once, PATO loads the input sequences in blocks and executes the search algorithms iteratively to limit the maximum memory that may be consumed by the application. Furthermore, PATO leverages efficient data structures as well as algorithms from the SeqAn 2.4.0 template library (Reinert et al. 2017).

Because new emerging triplex-centric pipelines work by calling Triplexator under the hood, PATO exports the same command line interface and output formats as Triplexator. Therefore, PATO’s adoption should be as easy as swapping Triplexator for PATO. Moreover, PATO allows a more relaxed set of prediction parameters, and it can search for triplex features of as little as five nucleotides in length.

With regard to minimum hardware requirements, PATO has none. It can be compiled and run on, but not limited to, x86, x86_64, and ARM processors (including M1-based systems). Main memory capacity is not a problem for PATO either. As it will be shown in Section 3, PATO can run triplex analyses against the human genome (60 GB of sequence data) using as little as 6.41 GB of memory.

## 3 Results

**Figure 1:**
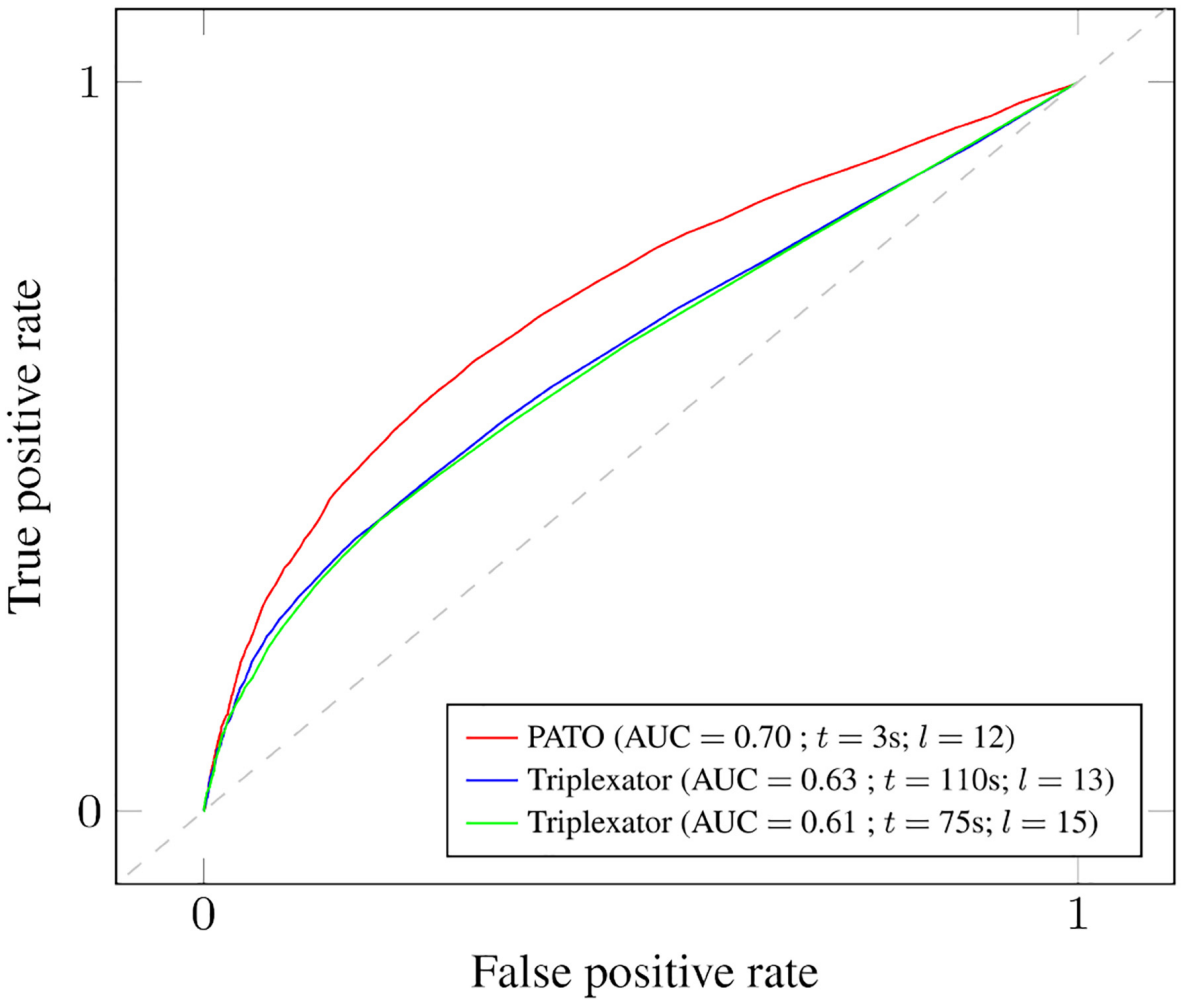
The ROC curves demonstrating the accuracy of the lncRNA–DNA triplexes predicted by PATO and Triplexator on the experimentally validated MEG3-bound genomic regions as in (Antonov et al. 2019). AUC, area under the curve; *t*, time spent searching for triplexes; *l*, minimum triplex length allowed

Table 1 shows the four genome-wide datasets selected to evaluate the performance capabilities of PATO. Table 2 and Fig. 1 present benchmark comparison results between PATO and Triplexator. We did not consider other triplex prediction tools as they proved to be much slower and less accurate than Triplexator in a recent study (Antonov et al. 2019).

**Table 1:** Genome-wide datasets used to assess the capabilities of PATO

Species	TFOs target size (MB)	TTSs target size (GB)
*Lepidothrix coronata*	0.7	1.1
*Ursus americanus*	1.0	2.5
*Anser brachyrhynchus*	12.0	1.1
*Homo sapiens*	79.0	60.0

**Table 2:** Performance comparison of Triplexator, PATO on a single core, and PATO run in parallel using two different computing environments^a^

Machine	Species	Triplexator	PATO (single core)			PATO (parallel)		
			Runtime	Sim. seqs.	Mem. usage (GB)	Runtime	Sim. seqs.	Mem. usage (GB)
Two-socket server	*Lepidothrix coronata*	47 min 47 s	4 min 58 s	128	1.33	22s	128	6.39
	*Ursus americanus*	4 h 6 min 37 s	23 min 34 s	128	0.17	1 min 26 s	128	0.88
	*Anser brachyrhynchus*	10 h 48 min 22 s	1 h 0 min 58 s	128	2.28	3 min 35 s	128	7.04
	*Homo sapiens*	7 days 15 h 54 min 21 s^b^	17 h 11 min 15s	128	25.52	55 min 13 s	128	197.52
Desktop computer	*Lepidothrix coronata*	33 min 56 s	3 min 4 s	16	0.47	40 s	16	2.23
	*Ursus americanus*	2 h 54 min 33 s	14 min 31 s	16	0.07	2 min 12 s	16	0.30
	*Anser brachyrhynchus*	7 h 36 min 48 s	35 min 50 s	16	0.59	5 min 25 s	16	2.55
	*Homo sapiens*	5 days 7 h 14 min 39 s^b^	10 h 10 min 46 s	4	6.41	1 h 45 min 2 s	2	9.70

a Two-socket server: 2 × Intel Xeon Silver 4216 (32 CPU cores/64 threads @ 2.10 GHz; Turbo: 3.20 GHz), 256 GB DDR4 main memory (8 × 32 GB), and GCC v9.3.0. Desktop computer: 1 × Intel i7-10870H (8 CPU cores/16 threads @ 2.20 GHz; Turbo: 5.00 GHz), 16 GB DDR4 main memory (2 × 8GB), and GCC v9.4.0. Search parameters: Triplexator’s defaults.

b The runtime is an estimate. It was computed taking into account PATO’s sequential runtime and the average speedup of PATO with respect to Triplexator.

On the one hand, Table 2 highlights the important differences in runtime between Triplexator, PATO on a single core, and PATO run in parallel when performing four genome-wide analyses on two different computing environments. A remarkable result from this table is that PATO, spawning 64 threads, is up to 200 times faster than Triplexator, and it finds triplex features over the entire human genome in under 1 h. Furthermore, Table 2 shows that PATO is not limited to high-end computing devices, and it can run genome-scale analyses in standard desktop computers too. This is possible thanks to the fact that our novel application can fine-tune the memory usage of its search algorithms by setting the maximum number of sequences that may be processed simultaneously (column ‘Sim. seqs.’ in the table). PATO’s user manual provides a tutorial and hints to set this number to a value that can get the most out of the user’s system. Triplexator’s parallel runtime was not considered since its embedded parallel run modes proved to be insufficient to accelerate the analyses featured in Table 2. Indeed, some runs ended up taking more time in parallel, and some others crashed abruptly midway through the triplex search pipeline. In order to make Table 2 a fair comparison, we had to manually patch Triplexator so that it could be compiled with an up-to-date C++ compiler. Thus, we do not only show that Triplexator is far slower than PATO, but we also show that Triplexator as it is requires legacy software to run on today’s computing environments.

Figure 1, on the other hand, evaluates the prediction performance of PATO and Triplexator using lncRNA MEG3 and the experimentally validated MEG3-bound genomic regions. The figure shows that PATO allows a more accurate and much faster search of the solution space than Triplexator. That is, as Matveishina et al. (2020) proved that lncRNA–DNA predictors perform the best when parameters aid the search towards small and degenerated (i.e. with high error rates) triplexes, tools must be able to search for this type of features in order to obtain the most accurate results. Lowering the minimum triplex length and allowing higher error rates, however, dramatically increases the runtime of the Triplexator. On the contrary, because PATO is significantly faster and more efficient than Triplexator, it can reduce the minimum triplex length without leading to prohibitive runtimes, meaning that PATO can explore the solution space with more precise prediction parameters and obtain more accurate results in far less time than Triplexator.

Conflict of interest: None declared.
